# Supplementation of L-Ornithine Could Increase Sleep-like Behavior in the Mouse Pups

**DOI:** 10.3390/metabo12121241

**Published:** 2022-12-09

**Authors:** Mayumi Takakura, Satsuki Nagamachi, Takuma Nishigawa, Yoshihiro Takahashi, Mitsuhiro Furuse

**Affiliations:** 1 Laboratory of Regulation in Metabolism and Behavior, Faculty of Agriculture, Kyushu University, Fukuoka 819-0395, Japan; 2 Faculty of Life Science, Kyushu Sangyo University, Fukuoka 813-8503, Japan

**Keywords:** L-ornithine, breast milk, pups, sleep-like behavior, polyamine, mouse

## Abstract

Along the maternal–fetal–neonatal axis, one of the problems relating to the maternal–neonatal axis is infant sleep problems including nighttime crying. One possible solution could be to provide the newborn with sleep-promoting ingredients through breast milk or formula. So far, it has been reported that L-ornithine has a sleep-related effect. Therefore, we investigated the effect of dietary L-ornithine on maternal mouse plasma and milk L-ornithine levels in Experiment 1. In Experiment 2, a single dose of L-ornithine was applied to know the time-course changes in plasma, mammary gland and milk L-ornithine levels. Experiment 3 was conducted to confirm sleep behavior as well as changes in polyamine levels in milk. L-Ornithine levels in maternal plasma significantly increased by both dietary regimen and single oral administration in Experiments 1 and 2. Both L-ornithine treatments also increased its levels in milk, although not to a concentration as high as in plasma. In Experiment 3, the level of polyamines, which are metabolized from L-ornithine, did not significantly differ after L-ornithine administration. In sleep-like behavior observations, the average concentration of L-ornithine in milk did not increase the sleep-like behavior of mouse pups. However, more concentrated L-ornithine solutions can significantly increase sleep-like behavior. These results revealed that even if mothers ingested L-ornithine to increase L-ornithine levels in breast milk, it is difficult to promote sleep in newborns. Because it is difficult to raise L-ornithine in breast milk to sleep-inducing levels, L-ornithine added formula may partially improve infant sleep and has the potential for preventing infant sleep problems such as nighttime crying.

## 1. Introduction

Nighttime crying is a serious problem for parents; infants often wake up and cry heavily late at night. The groups of people who display nighttime crying include infants, toddlers, and children [1]. According to a study conducting a questionnaire to mothers, half of infants wake up more than four nights per week, and more than a third wake up every night [2]. Continuous nighttime crying may become a big stressor and a heavy burden for parents. In fact, when asked, parents answered that they felt depression or sleep deprivation because of infant sleep problems [2,3]. This continuous burden could lead to family dysfunction, including child neglect or abuse [3]. Therefore, it is important to find a way to reduce nighttime crying such that mental and physical stresses are reduced and both parents and infants live healthily.

Nighttime crying is a recognized problem for parents; however, its mechanism is little known. Rapid eye movement (REM) sleep and non-rapid eye movement (NREM) sleep are known as fundamental sleep states. In general, NREM sleep is a deep sleep, while REM sleep is a light sleep; animals, such as mammalian and avian species, switch between these two states [4]. One cycle of human sleep takes about 90 min in adults, but it is shorter in infants, around 50 to 60 min long [5]. Therefore, REM sleep is more frequently seen in infants than in adults. Moreover, the ratio of REM sleep to total sleep in neonates is higher than that in adults [6]. According to Burnham et al. [7], infants who exhibit high levels of NREM sleep earlier in life are likely to exhibit the desired outcome of self-soothing, an infant’s ability of calming from crying to quiet wakefulness without parental assistance. Considering these reports, infants could wake up easier than adults even from a deep sleep state; thus, we assumed that promoting NREM sleep could reduce the occurrence of nighttime crying.

There are many ways to improve sleep. One of them is to take a medicine; hypnotic drugs effectively exert their effects on people who have a sleep-related disease. However, drugs might have side effects and are not always safe for infants. Moreover, most parents are reluctant to use drugs on their infants, even when they would like to make them sleep deeply. Currently, herbal medicines are sold in Japan to prevent night crying, but we hope that in the future, things such as amino acids that are required by the body or produced in the body will be able to reduce night crying. It has been shown that some amino acids can improve sleep [8]. These functional amino acids are contained in common food and have fewer side effects. Among them, L-ornithine is a non-proteinogenic amino acid commonly contained in foods such as corbicula, mushrooms, and cheese. It has been reported that L-ornithine has both sedative and hypnotic effects in neonatal chicks [9,10] and an anxiolytic-like effect in mice [11]. L-Ornithine also promotes NREM sleep in mice [12] and improves sleep quality in humans [13]. Therefore, infant supplementation with L-ornithine could promote infant deep sleep and lead to reduce nighttime crying. However, the studies that have been conducted so far have been limited to adult experimental animals and adults, and there are no studies on infants. Furthermore, there have been few attempts to simultaneously induce sleep in mothers and infants in response to night crying.

Breast milk is the sole nutritional source for infants of mammalian species, implying that milk is the most preferable way of supplying L-ornithine to infants. Breast milk constituents are synthesized from three different sources. The mammary secretory cells synthesize some of these components from precursors in the plasma, while some nutrients are produced by other cells in the mammary glands. Finally, other constituents are transferred directly from plasma to milk [14]. Blood constituents are changed by maternal meal ingestion, implying that it might be possible to change the milk constituents and add desired functions to breast milk by altering the maternal diet. Therefore, certain functional nutrients in breast milk, controlled by maternal meal ingestion, could affect the development or behavior of the infant. In other words, the presence of L-ornithine in milk, controlled by the maternal diet, could improve infant sleep. However, mouse milk contains L-amino acid oxidase, a lactating mammary gland-specific protein. Consequently, the content of free amino acids targeted by L-amino acid oxidase was very low in conventional milk [15]. For instance, the L-arginine concentration in the milk showed a meager increase of 5 nmol/mL in the L-arginine administration group, while it was significantly increased by about 150 nmol/mL in the plasma as compared to the control group [16]. On the other hand, although L-serine levels did not change significantly in the plasma of the L-serine-treated group, their levels in maternal milk were high (more than 100 nmol/mL) compared with the control group. These data suggested that plasma L-serine transferred efficiently to milk [17]. However, no report has so far been made on the transfer of L-ornithine to milk.

In this study, we aimed to investigate the possibility of enhancing the concentration of L-ornithine in breast milk as a solution for nighttime crying by documenting the transfer of L-ornithine from the maternal body into milk. In addition, the effect of L-ornithine from breast milk level to pharmacological level on mouse pups’ sleep-like behavior was investigated to clarify how much L-ornithine is required to induce sleep in newborn mice.

## 2. Materials and Methods

### 2.1. Animals

All animals used in this study were purchased from Japan SLC (Hamamatsu, Japan). For Experiment 1, male and female ICR mice were purchased and their eight-week-old offspring, 21 virgin females and fifteen males were used. For Experiment 2, 12 male and 30 female ICR mice (eight-week-old) were used. In Experiment 3, 23 pregnant ICR mice were used for milk sampling and twenty pups from mother mice in the control group were used for sleep-like behavior observations. Experiments 1 and 2 were carried out for the following reasons. Experiment 1 aimed to investigate the effect of dietary L-ornithine on maternal mouse plasma and milk L-ornithine levels. Experiment 2 used a single dose of L-ornithine to determine the time-course changes in plasma, mammary gland and milk L-ornithine levels. These data were also necessary to determine the administration level of Experiment 3. Experiment 3 was an experiment from a different viewpoint that was conducted to confirm sleep behavior. It was also necessary to know whether the change in L-ornithine was due to its metabolism to polyamines. Polyamines could also be measured in Experiment 2, but it was not possible due to the amount of sample. Therefore, the polyamine level was measured in Experiment 3. All mice were maintained at a temperature of 23 ± 1 °C on a 12 h light/dark cycle (lights on at 8:00 a.m. and lights off at 8:00 p.m.). Food (MF; Oriental Yeast, Tokyo, Japan) and tap water were given ad libitum. All animal experiments reported here were conducted in accordance with the Guidelines for Animal Experiments in the Faculty of Agriculture at Kyushu University (A28-069-4), as well as law No. 105 and Notification No. 6 from the Japanese Government.

### 2.2. Experimental Procedures

In Experiment 1, seven female mice and five male mice were paired in the same cage. Female mice were separated individually on the 11th day after pairing. Mothers were divided into two groups (control and ornithine group) on the day of parturition (postnatal day 0, P0). Milking was conducted at P9. Blood samples were obtained both from the mother and her pup under anesthesia with isoflurane (Escain^®^, Mylan, Osaka, Japan) at P21 and P12, respectively. At P0, mice from the ornithine group were given the powdered diet supplemented with L-ornithine hydrochloride (Kyowa Hakko Bio Co., Ltd., Tokyo, Japan) to a final concentration of 2% (wt/wt) L-ornithine. Mice in the control group were continuously given the standard diet. Plasma and milk samples were stored at −80 °C until analysis took place.

For Experiment 2, groups of five to six female mice and two males were paired in the same cage. Then, female mice were individually separated on the 11th day after pairing. At P0, mothers were divided into six groups: administration of L-ornithine 1 h (n = 5), 2 h (n = 5), 3 h (n = 5), 4 h (n = 5), and 5 h (n = 5) before milking, and the control group (n = 5). At P9, milking was conducted after an oral administration of L-ornithine: L-ornithine hydrochloride was dissolved in distilled water to a final concentration of 3.0 mmol (400 mg)/10 mL/kg. Mice were euthanized under isoflurane anesthesia, and blood and mammary gland samples were obtained.

In Experiment 3, twenty-three mothers were divided into four groups at P0: administration of L-ornithine 1 h (n = 5), 2 h (n = 5), and 3 h (n = 5) before milking, and control (n = 8). Milking was done following the same procedure used in Experiment 2. At P14-16, sleep-like behavior observations were conducted for twenty pups from mothers in the control group. L-Ornithine monohydrochloride was dissolved in distilled water to make solutions to concentrations of 3.0 mmol ornithine/10 mL/kg for mother mice and 0.1 mg ornithine/10 mL/kg, 126 mg ornithine/10 mL/kg, and 2.0 g ornithine/10 mL/kg for pups.

### 2.3. Milking

Milking was done according to Nagamachi et al. [17]. Milk was stored at −80 °C until analysis.

### 2.4. Amino Acid and Polyamine Analysis

The concentration of L-ornithine in each sample was analyzed according to a previously described method [18]. The concentration of polyamines, putrescine, spermidine, and spermine in milk was measured by high-performance liquid chromatography (HPLC) following a previously reported method [19] with some modifications. For sample preparation, milk was transferred into ultrafiltration tubes (Millipore, Bedford, MA, USA) and centrifuged at 14,000× *g* at 4 °C for 20 min to deproteinize it, and the filtrate was recovered. 1,6-Diaminohexane (100 µM, 3 µL) was added to 47 µL of a milk sample or standard solution as an internal standard. Then, 300 µL of dansyl chloride dissolved in acetone (10 mg/mL) and 50 µL of saturated sodium carbonate were added to 50 µL of the above solution. The solution was then mixed and left overnight at room temperature under dark conditions. Then, 50 µL of a proline solution (100 mg/mL) was added and left for 30 min at room temperature. Toluene (500 µL) was added and vortexed. After it was centrifuged, 700 µL of the upper layer was taken and the solvent was eliminated by using nitrogen gas. Dried residues were dissolved in 50 µL of an acetonitrile–water solution (1:1) and used for our analysis. The levels of polyamines (putrescine, spermidine, and spermine) were measured by Prominence-i apparatus (Shimadzu, Kyoto, Japan) with a TSKgel ODS-80Ts 4.6 × 250 mm column (Tosoh, Tokyo, Japan). The excitation and emission wavelengths for fluorescence detection were 340 nm and 515 nm, respectively. The system was operated with a flow rate of 1 mL/min at a column temperature of 40 °C. The injection volume was 20 µL. HPLC gradients (solvent A = water, solvent B = 100% acetonitrile) were 60% B for 10 min, 60–100% B for 20 min, 100% B for 15 min, and 60% B for 10 min.

### 2.5. Sleep-like Behavior Observation

In Experiment 3, sleep-like behavior observation was conducted for pups at P14-16. On the day of the observation, each pup was moved from its home cage to an observation cage one hour before the beginning of the observation. Then, either (1) water, as control; (2) 0.1 mg ornithine/10 mL/kg, the average milk level from results of Experiment 2; (3) 126 mg ornithine/10 mL/kg, the anti-anxiety effect concentration reported by Kurata et al. [11]; or (4) 2.0 g ornithine/10 mL/kg, the concentration needed for an NREM sleep increase reported by Omori et al. [12] was administered to each pup before returning it to its observation cage. Pups’ behavior was recorded for 2 h by video cameras (I-O DATA DEVICE, Kanazawa, Japan). Behavior was regarded as sleep-like when a mouse sat motionless with its eyes closed and its head dropped. Sleep latency, longest sleep duration, total sleep time, sleep episodes and mean sleep duration were investigated during observation.

### 2.6. Statistical Analysis

Data from each experiment were subjected to Thompson’s rejection test to eliminate outliers (*p* < 0.01); the remaining data were then used for the analysis. In Experiment 1, L-ornithine levels between control and ornithine groups were analyzed by *t*-test. A Mann–Whitney U-test was applied to data that did not have a normal distribution or homoscedasticity. In Experiments 2 and 3, data were analyzed either by a one-way ANOVA or by a Kruskal–Wallis test if the data had homoscedasticity or heteroscedasticity, respectively. When significance was detected in these tests, either a Tukey–Kramer test or a Steel–Dwass test was applied as a post hoc test. Moreover, regression analysis was applied to the data of pup sleep-like behavior in Experiment 3. All analyses were performed with the StatView software (version 5, SAS Institute, Cary, NC, USA, SAS 1998) except Thompson’s rejection test (*p* < 0.01) and the Steel–Dwass test. Thompson’s rejection test was performed with JSTAT (version 11.1) and the Steel–Dwass test was done by Statcel3 (The Publisher OMS Ltd., Saitama, Japan, 2011). Significance was set at *p* < 0.05 and a tendency was set at *p* < 0.1. Data were expressed as mean ± SEM.

## 3. Results

### 3.1. Concentration of L-Ornithine in Mother Plasma and in Milk

**Figure 1:**
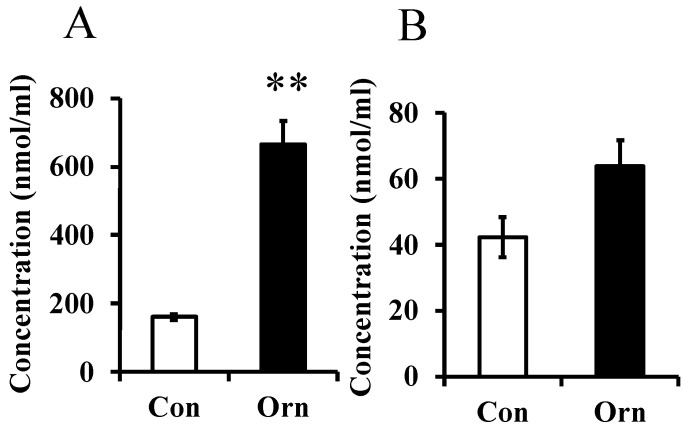
Concentration of L-ornithine in mother plasma at P21 (**A**) and in milk at P9 (**B**) in Experiment 1. Data are expressed as means ± SEM. n = 6–8/group. ** *p* < 0.01. Con: control, Orn: ornithine.

In Experiment 1, the level of L-ornithine in maternal plasma was greatly increased by dietary L-ornithine as shown in Figure 1A. L-Ornithine concentration in milk, lower than that in plasma, tended to increase in the ornithine group (*p* = 0.0507; Figure 1B). However, the L-ornithine level in the pup’s plasma was not significantly different between the two groups (data not shown).

### 3.2. Levels of L-Ornithine in Maternal Plasma, the Mammary Glands, and Milk after Oral Administration of L-Ornithine

**Figure 2:**
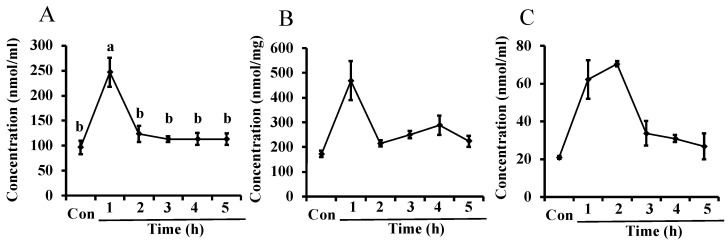
Levels of L-ornithine in maternal plasma (**A**), the mammary glands (**B**), and milk (**C**) after oral administration of L-ornithine in Experiment 2. Data are expressed as means ± SEM. n = 3–5/group. Con: control. In (**A**), the 1 h value marked with the letter “a” is significantly higher than the other values marked with the letter “b” at *p* < 0.05.

In Experiment 2, L-ornithine levels in maternal plasma significantly increased 1 h after administration of L-ornithine compared to the control but decreased to pre-administration levels after 2 h (Figure 2A). The levels of L-ornithine in the mammary glands and milk (Figure 2B,C, respectively) were significantly different between experimental and control groups in the Kruskal–Wallis test, but no significant difference was seen after a Steel–Dwass test was conducted as post hoc test. The levels of L-ornithine in the mammary glands increased 1 h after L-ornithine administration and then returned to pre-administration levels, similar to the change in plasma. L-Ornithine levels in milk increased after L-ornithine administration and reached a peak 1–2 h before decreasing.

### 3.3. Polyamine Levels in Milk after Oral Administration of L-Ornithine

**Figure 3:**
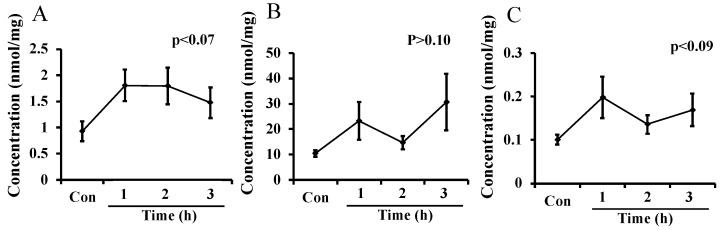
Putrescine (**A**), spermidine (**B**), and spermine (**C**) levels in milk after oral administration of L-ornithine in Experiment 3. Data are expressed as means ± SEM. n = 4–8/group. Con: control.

The levels of putrescine, spermidine, and spermine in breast milk, measured in Experiment 3, are shown in Figure 3A–C, respectively. The level of spermidine was the highest and that of spermine was the lowest in mice milk among the three polyamines. The level of each polyamine in milk tended to increase 1 h after administration of L-ornithine, but not significantly. The changes in polyamine levels were not much larger than the difference in L-ornithine levels between maternal plasma and milk seen in Experiments 1 and 2.

### 3.4. Sleep-like Behavior of Mouse Pups Supplemented with L-Ornithine at Different Concentrations

**Figure 4:**
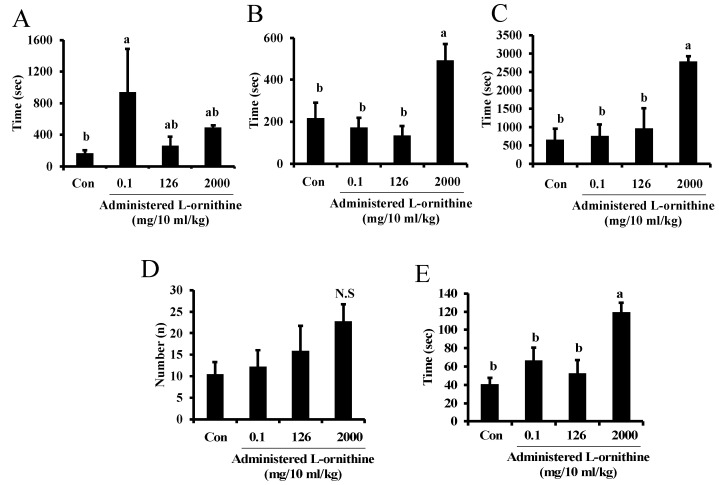
Sleep-like behavior of mouse pups supplemented with L-ornithine at different concentrations. Sleep latency (**A**), the longest sleep duration (**B**), total sleep time (**C**), sleep episode (**D**), and mean sleep duration (**E**) in pups at P14-16 in Experiment 3. Data are expressed as means ± SEM. n = 4–5/group. Con: control. The value marked with the letter “a” is significantly higher than the other values marked with the letter “b” at *p* < 0.05. If having common letters (a vs. ab or b vs. ab), it is not significantly different. N.S indicates no significant difference from control.

Figure 4 shows sleep latency (A), the longest sleep duration (B), total sleep time (C), sleep episode (D) and mean sleep duration (E) in pups at P14-16. Sleep latency significantly increased in the second group compared to the control group. Longest sleep duration, total sleep time and mean sleep duration were significantly higher in the fourth group, while low concentrations of administered L-ornithine to the second and third groups had no significant effects on them. Sleep episode was not significantly different among groups. In regression analysis, no significant correlation was seen between administered L-ornithine concentration and sleep latency, whereas there were positive correlations between administered L-ornithine levels and longest sleep duration (F(1, 18), R^2^ = 0.497, *p* < 0.001), total sleep time (F(1, 17), R^2^ = 0.576, *p* < 0.001), sleep episode (F(1, 18), R^2^ = 0.206, *p* < 0.05) and mean sleep duration (F(1, 17), R^2^ = 0.552, *p* < 0.001).

## 4. Discussion

Plasma L-ornithine concentration hardly influenced the levels of L-ornithine in milk even though plasma L-ornithine concentration in mothers was greatly enhanced by dietary L-ornithine supplementation in Experiment 1. Thus, it was hypothesized that plasma L-ornithine was difficult to transfer to milk. Moreover, L-ornithine metabolism might be affected by an unknown mechanism at the mammary glands or, alternatively, might occur within the mammary gland itself. It was also considered that the collection time of both milk and maternal plasma might not match. In Experiment 1, mothers and pups were separated 5 h before milking. However, we did not follow up the eating times of mother mice during these 5 h; thus, milk might not have been collected at its highest level of L-ornithine. To clarify the effect of time lag of sampling for plasma and milk, acute administration of L-ornithine was conducted in Experiment 2.

Data from Experiment 2 showed that L-ornithine level in maternal plasma rapidly increased and reached its highest concentration 1 h after administration, but returned to pre-administration level after 2 h. L-Ornithine levels in the mammary glands showed a similar pattern. The levels of L-ornithine in the plasma of the ornithine groups differed between Experiments 1 and 2. The highest level of L-ornithine in maternal plasma in Experiment 2 was lower than that of the L-ornithine group in Experiment 1. Nevertheless, it was hard to compare the results from the two experiments because the amount of L-ornithine eaten by mice before sampling in Experiment 1 was unclear; it can be hypothesized that L-ornithine reached its peak before 1 h and had already decreased by the time at which sample was taken. In fact, Omori et al. [12] reported that L-ornithine level was increased in rat serum 10 min after ingestion and reached its highest peak after 50 min.

It is considered that the milk L-ornithine level increased due to orally administered L-ornithine; its peak was reached 1–2 h after administration of L-ornithine. Notably, this peak appeared later than concentration peaks from plasma and the mammary glands. A transfer like this has also been reported for humans [20]. The highest peak of concentration of L-ornithine was not stable, it decreased soon after, maybe by the action of the L-amino acid oxidase. Nagaoka et al. [15] reported that free amino acids in mouse milk were metabolized in the mammary glands, since the activity of L-amino acid oxidase was high. L-Arginine transfer to milk is certainly low [16], but L-ornithine, a metabolite of L-arginine investigated here, seemed to transfer to milk more easily than L-arginine. This is close to the results observed with L-serine [17]. Our results from the time-course changes in L-ornithine concentration suggest that the time of L-ornithine ingestion by the mother may be important to give L-ornithine-rich milk to the infant. The highest levels of L-ornithine in milk were similar between Experiments 1 and 2, and continuous administration might not be the reason for the big difference observed in L-ornithine levels between milk and plasma in Experiment 1.

L-Ornithine is metabolized to polyamines in the mammary glands. Polyamines are contained in milk and have some effects on the maturation of the infant’s alimentary canal and on the promotion of infant development [21]. In addition, it was reported that polyamines regulate the activation of the N-methyl-D-aspartate (NMDA) receptor and are related to learning and memory in mammals [22]. Consequently, if the concentration of polyamines in milk from the ornithine group were higher than that in the control group, L-ornithine supplementation to the mother might affect pups’ learning and memory. L-ornithine might play an important role as a precursor of polyamines in breast milk. Accordingly, in Experiment 3, polyamine levels in milk and pup sleep-like behavior were investigated. Putrescine is synthesized from L-ornithine by the action of ornithine decarboxylase. Ornithine decarboxylase expression in the mammary glands increased during lactation [23] and many studies reported that milk contains polyamines. Spermidine is synthesized from putrescine and decarboxylated S-adenosylmethionine by the action of spermidine synthase, and spermine is synthesized from spermidine and decarboxylated S-adenosylmethionine by the action of spermine synthase. It was assumed that L-ornithine decreases in milk due to the synthesis of polyamines. As a result, the concentration of polyamines including putrescine, spermidine, and spermine should have a tendency to increase in milk after L-ornithine administration to the mother. As expected, each polyamine level tended to increase 1 h after L-ornithine administration. From the results of Experiment 2, it was found that L-ornithine reached mammary glands 1 h after administration, suggesting that polyamine metabolism from orally administered L- ornithine to putrescine can start rapidly. Polyamine levels in milk were high for spermidine, lower for putrescine, and even lower for spermine, as previously reported for rat milk [24]. In human milk, however, it was found that putrescine level was very low compared with spermidine or spermine [24,25], suggesting that the concentration of each polyamine would differ between species after L-ornithine administration. In contrast, Harada et al. [19] reported different polyamine levels in mouse skin, being higher for spermidine, lower for spermine, and even lower for putrescine, suggesting that polyamine metabolism must be tissue specific. The putrescine level in milk was much lower than that of spermidine, and the change in spermidine levels was much larger than that of putrescine, implying that putrescine was produced at a high rate by the action of ornithine decarboxylase, but rapidly converted into spermidine in the mammary glands. Spermidine is then metabolized into spermine by the action of spermine synthase. As the level of spermine was quite low in mouse milk, it was assumed that the activity of spermine synthase in the mammary glands might be low, too. From these results which show that the changes in each polyamine level were not much larger than the difference in L-ornithine levels between maternal plasma and milk, it can be concluded that low milk L-ornithine levels compared with those from plasma cannot be solely explained by L-ornithine metabolism to polyamines in the mammary glands.

Regression analyses showed a positive correlation between administered L-ornithine levels and every sleep-like behavior parameter except for sleep latency. Particularly, administered L-ornithine levels strongly correlated with the longest sleep duration, total sleep time and mean sleep duration. The results suggest that a large amount of L-ornithine is needed to increase sleep-like behavior, indicating that L-ornithine levels in milk can hardly induce sleep. In our system, sleep in mouse pups cannot be measured by electroencephalogram and myoelectricity. Sleep estimated by behavioral observation alone was preliminary. Thus, we cannot distinguish it from the sedative effect. Anyway, L-ornithine supplementation could reach pharmacological levels. In the present study, only the highest level (2.0 g ornithine/10 mL/kg) of L-ornithine induced sleep in offspring. This ornithine level increased the amount of NREM sleep without reducing the power spectrum density of NREM sleep in mice [12]. In a human study, it was also confirmed that L-ornithine (400 mg ornithine/day) for 8 weeks has a positive effect on stress and sleep in healthy workers [13]. It can be seen that the effective concentration of L-ornithine for sleep was considerably lower in humans than in mice. It is currently unknown how much L-ornithine is absorbed by human mothers and is transferred into breast milk. If it is transferred effectively, it may be possible to improve sleep through breast milk in humans. In addition, although it is necessary to confirm the safety, adding a low dose of L-ornithine to infant formula may partially lead to a solution to the problem of crying at night.

## 5. Conclusions

L-Ornithine levels in maternal plasma were significantly increased by both dietary regimen and single oral administration. Both L-ornithine treatments also increased its levels in milk, although not to a concentration as high as in plasma. The level of polyamines, which are metabolized from L-ornithine, did not significantly differ after L-ornithine administration. In sleep-like behavior observations, the average concentration of L-ornithine in milk did not increase sleep-like behavior in mouse pups. However, more concentrated L-ornithine solutions can significantly increase sleep-like behavior. These results revealed that even if mothers ingested L-ornithine to increase L-ornithine levels in breast milk, it was difficult to promote sleep in pups. Because it is difficult to raise L-ornithine in breast milk to a sleep-inducing level, L-ornithine added to infant formula may improve infant sleep and has the potential to reduce infant sleep problems such as nighttime crying. However, since the dose of L-ornithine that affects sleep differs considerably between the mice used in this study and humans, the sleep-improving effect of L-ornithine via human breast milk or addition to infant formula should be examined while considering safety.

## Data Availability

Data are contained within the article.
